# Balancing Hydrogen Bonding and Crystallinity in Enzymatically Functionalized Lignin–Poly(vinyl alcohol) Composite Films

**DOI:** 10.3390/polym18182238

**Published:** 2026-09-14

**Authors:** Weijun Liang, Gabriela Dominguez, Miguel Panizo-Laiz, Olga Martin, Alberto García-Peñas

**Affiliations:** 1 Departamento de Ciencia e Ingeniería de Materiales e Ingeniería Química, Universidad Carlos III de Madrid, IAAB, Avenida Universidad 30, 28911 Leganés, Spain; wliang@ing.uc3m.es; 2 Departamento de Biomedicina y Biotecnología, Facultad de Farmacia, Universidad de Alcalá, Ctra. Madrid-Barcelona km 33.6, 28871 Alcalá de Henares, Spain; gabriela.dominguez@uah.es; 3 Departamento de Física Aplicada e Ingeniería de Materiales, Escuela Técnica Superior de Ingenieros Industriales de Madrid, UPM, Calle José Gutiérrez Abascal, 2, 28006 Madrid, Spain; miguel.panizo.laiz@upm.es

**Keywords:** PVA, functionalized acid soluble lignin, hydrogen bonding, nanoindentation, tensile strength

## Abstract

Poly(vinyl alcohol) (PVA) is a promising biodegradable polymer; however, its reliance on petroleum and incompatibility with raw lignin additives hinder sustainable PVA composite development. In this study, mechanically robust PVA/functionalized acid-soluble lignin (fLGAS) composite films were developed by means of aqueous solution casting without toxic chemical crosslinkers. Commercial kraft lignin was enzymatically modified using laccase from *Coriolopsis* spp., improving its compatibility with the PVA matrix. Molecular characterization confirmed the formation of an intermolecular hydrogen-bonding network between PVA and fLGAS, which significantly enhanced the thermal stability of the composites. Additionally, this network improved moisture response, achieving complete surface wetting (water contact angle decreasing from 47.04° to 0°) and swelling ratios exceeding 250% after 2 h. Nanoindentation and dynamic mechanical analysis characterizations revealed that mechanical properties enhanced with fLGAS loading up to 5 wt%, while the overall properties were governed by the balance between hydrogen bonding and crystallinity. This work provides an eco-friendly framework for industrial lignin valorization and sustainable functional film design.

## 1. Introduction

Poly(vinyl alcohol) (PVA) is an extensively utilized synthetic polymer favored for its excellent biocompatibility, biodegradability, and ease of chemical processing and functional modification [1,2]. However, the exclusive reliance on petroleum-derived PVA presents ongoing environmental and sustainability challenges [3]. Replacing a portion of synthetic PVA with renewable natural biopolymers serves a dual purpose: reducing petroleum-based polymer consumption and valorizing industrial biowaste into high-performance composite films [4,5]. Beyond chemical modification, constructing physical cross-linking networks via hydrogen bonding between functionalized biopolymers and PVA hydroxyl groups has emerged as an environmentally benign and scalable strategy. These physical interactions can restrict chain sliding, thereby enhancing structural stability without the need for toxic chemical cross-linkers (e.g., glutaraldehyde or epichlorohydrin) [6].

Among plant-derived biopolymers, lignin is a major by-product of the paper industry [7], which is typically released as waste or burned to produce heat [8]. Although conventional depolymerization strategies primarily target the cleavage of C–O bonds, more than 60% of lignin’s complex structure consists of robust C–C linkages, resulting in an overall lignin utilization rate of less than 2% [9]. Nevertheless, lignin is primarily composed of hydroxyphenyl (H), guaiacyl (G), and syringyl (S) units, which provide abundant functional groups capable of promoting physical cross-linking through hydrogen bonding [10]. Despite considerable efforts to exploit lignin’s unique properties and address environmental concerns, poor interfacial compatibility between PVA and lignin remains a major challenge in the development of high-performance composite films [11,12].

To overcome the aforementioned incompatibility and fully exploit lignin’s reinforcing potential, tailored modification of its chemical structure is essential. In this context, thermochemical and biological pretreatments represent the two primary strategies for lignin modification. Compared with thermochemical processes, biological approaches, including microbial and enzymatic treatments, offer greater selectivity and are more environmentally benign [13,14].

In this study, we propose a green, sustainable strategy for valorizing industrial waste lignin by partially replacing synthetic PVA with laccase-functionalized acid-soluble lignin (fLGAS) to fabricate high-performance, biodegradable composite films via a chemical-free physical blending route. Laccase derived from *Coriolopsis* spp. was employed as an enzymatic modification approach to regulate lignin side-chain structures and functional group content, thereby enhancing its dispersibility in aqueous and natural solvent systems. The improved solubility and compatibility of fLGAS enabled its uniform distribution within the PVA matrix, contributing to enhanced mechanical performance of the resulting composites. Furthermore, by achieving an optimal balance between intermolecular hydrogen bonding and matrix crystallinity, we revealed the fundamental structure–property relationships governed by the delicate equilibrium between these two competing forces. Additionally, the fluid absorption and swelling behavior of the resulting fLGAS/PVA films were systematically evaluated as standard material metrics to assess matrix continuity and network stability. This work offers a scalable, eco-friendly framework for high-value lignin utilization and advances the molecular design of uncrosslinked biopolymer composite films.

## 2. Materials and Methods

### 2.1. Materials

Polyvinyl alcohol (PVA, Mw: 89,000–98,000, 99+% hydrolyzed, Sigma-Aldrich, St. Louis, MO, USA), kraft lignin (Sigma-Aldrich, St. Louis, MO, USA), NaOH (Sigma-Aldrich, St. Louis, MO, USA), HCl (Sigma-Aldrich, St. Louis, MO, USA), and phosphate buffer (Sigma-Aldrich, St. Louis, MO, USA) were used as received. The enzyme was synthesized and cultivated at the Universidad de Alcalá (UAH), Madrid, Spain.

### 2.2. Preparation, Characterization and Properties

#### 2.2.1. Preparation of Functionalized Acid Soluble Lignin, fLGAS

The fLGAS was prepared as previously described [15]. Briefly, commercial kraft lignin was dissolved in 0.1 M NaOH for 24 h and subsequently acidified to pH 1–2 using 12 M HCl. The acid-soluble fraction of kraft lignin (kLGAS) was obtained via centrifugation (2000 rpm, 10 min) followed by filtration. The enzymatic reaction was prepared in a phosphate buffer (100 mM, pH 8), containing laccase derived from *Coriolopsis rigida* (1 U/mL of lignin) and the resulting acid-soluble fraction. The mixture was incubated for 24 h at 40 °C. The final pH of the sample was adjusted to pH 5 with 100 mM phosphate buffer. The final fLGAS was collected after freeze-dying with a Christ Alpha 1–4 freeze-dryer equipped with an LDC-1 M controller (Martin Christ, Osterode am Harz, Germany).

#### 2.2.2. Preparation of PVA/fLGAS Composite Films

**Figure 1:**
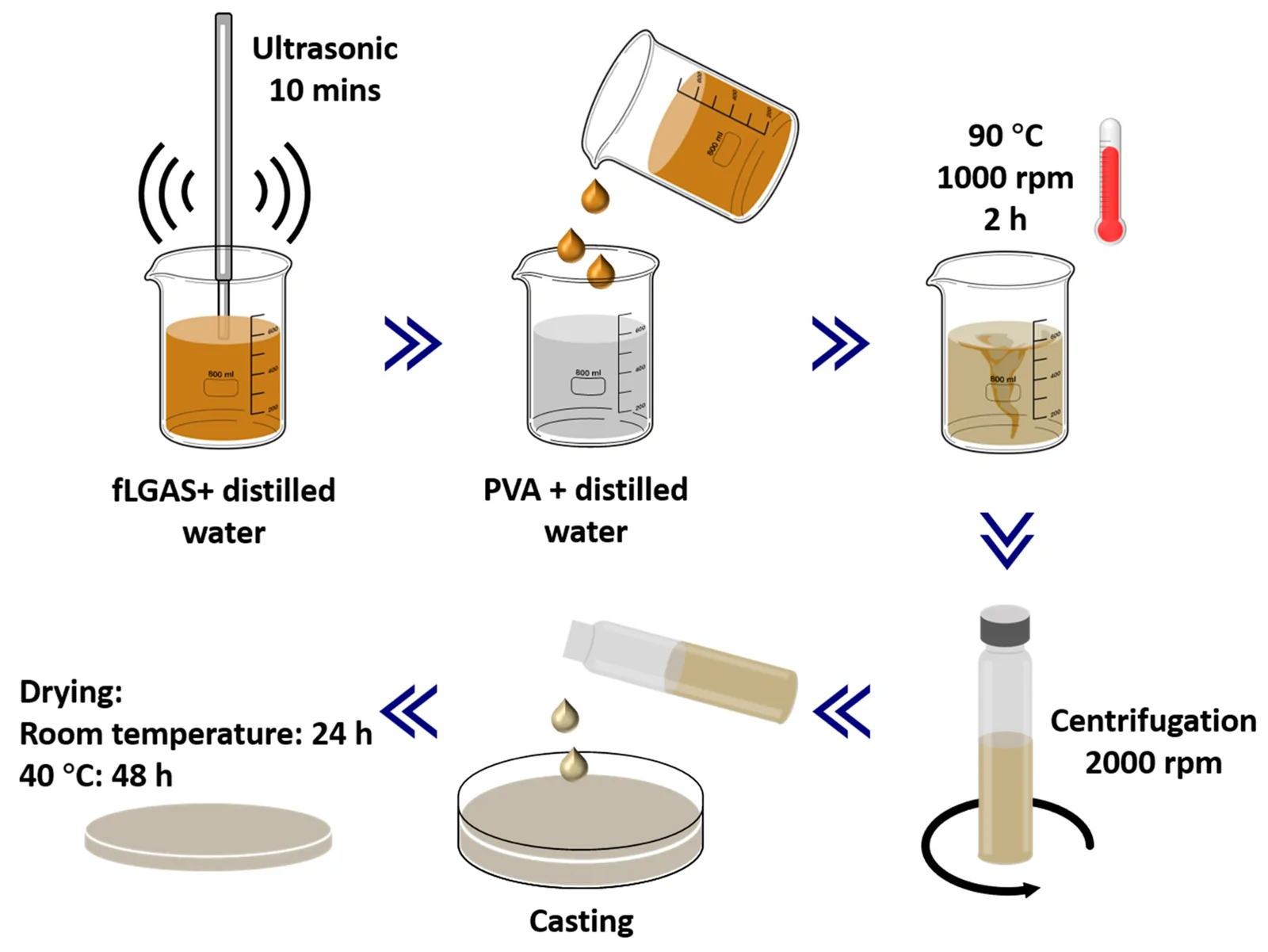
Schematic illustration of the casting process for PVA/fLGAS composite films. The curved lines represent ultrasonication, and the circular arrow indicates centrifugation.

Composite films were prepared using a solution-casting method with distilled water as the solvent (Figure 1). Briefly, fLGAS was dispersed in a portion of distilled water via ultrasonic treatment for 10 min (10 s on/30 s off cycles). The particle dispersion was then combined with the PVA solution, and the mixture was heated at 90 °C under continuous stirring at 1000 rpm for 2 h. After cooling to room temperature, the solution was centrifuged at 2000 rpm to remove entrapped air bubbles prior to casting. A total of 6.5 g of the solution was poured into Petri dishes (90 mm diameter), dried at room temperature for 24 h, and then placed in an oven at 40 °C for 48 h to obtain the composite films. The final film thickness was 0.055 ± 0.015 mm. The compositions of the PVA blend composite films are listed in Table 1. To evaluate the reinforcement capacity and processing bounds of fLGAS, a two-phase loading strategy was designed: a low-loading regime (0.1 and 0.5 wt%) to assess the dispersibility threshold and establish baseline nanofiller behavior, and a high-loading regime (5.0 and 10.0 wt%) to probe saturation.

The composite films are denoted using the format P-*x*fLGAS, where “P” represents the PVA matrix. The numerical prefixes represented by *x* indicate the weight percentages (wt%) of fLGAS, relative to the total mass of the composite. For instance, the P-5fLGAS refers to a ternary composite comprising 5 wt% fLGAS, with the remaining balance consisting entirely of PVA.

#### 2.2.3. Characterization and Properties

Fourier transform infrared (FTIR) spectroscopy was performed using an FTIR spectrometer (Jasco FT 6X, JASCO Corporation, Hachioji, Tokyo, Japan) equipped with an ATR PRO ONE (JASCO Corporation, Hachioji, Tokyo, Japan) single-reflection attenuated total reflection (ATR) accessory.

The morphological characterization was performed using a field-emission scanning electron microscope (FESEM, Hitachi SU-70, Chiyoda, Tokyo, Japan). For composite analysis, the accelerating voltage was 10 kV.

The composite films were characterized by X-ray diffraction (XRD) using a Malvern Panalytical Empyrean diffractometer (Malvern Panalytical, Almelo, The Netherlands) operated with Ni-filtered Cu Kα radiation (λ=1.54178 Å), collecting data over a 2θ range of 5–40°.

Thermal characterization was measured via differential scanning calorimetry (DSC) and thermogravimetric analysis (TGA). The thermograms were studied using a METTLER TOLEDO DSC 822e (Greifensee, Zürich, Switzerland). To eliminate the thermal history of the composite films, two heating-cooling cycles were performed for all samples under a nitrogen atmosphere. The thermal treatment was carried out between 25 and 300 °C at a constant heating and cooling rate of 10 °C/min. Thermal stability and decomposition behavior were evaluated by a TGA (PerkinElmer model STA 6000, PerkinElmer, Waltham, MA, USA). A heating cycle from 50 to 600 °C at 30 °C/min was used. The tests were conducted in an alumina boat under a nitrogen flow rate of 20 mL/min.

The swelling ratio was determined using the gravimetric method. The initial dry weight of the samples was recorded as $W_{\mathrm{dry}}$. The samples were then immersed in distilled water at room temperature for a specific duration until swelling equilibrium was reached. At predetermined time intervals, the samples were removed from the medium, and the excess surface water was gently blotted with filter paper before weighing $W_{\mathrm{wet}}$. The swelling ratio was calculated according to the following Equation (1):

(1) $$\mathrm{Swelling}\;\mathrm{Ratio}\;=\;\frac{W_{\mathrm{wet}}\;-\;W_{\mathrm{dry}}}{W_{\mathrm{dry}}}\;\times \;100\%$$

Water contact angle (WCA) was evaluated by measuring the static water contact angle using a Dataphysics OCA 15 plus contact angle goniometer (DataPhysics Instruments, Filderstadt, Germany) via the sessile drop method. At room temperature, a 2 μL droplet of distilled water was deposited onto the sample surface using a syringe.

Nanoindentation (DSI) measurements were conducted at 20 °C with a Shimadzu tester (model DUH211S, Shimadzu Corporation, Kyoto, Japan) equipped with a Berkovich type diamond indenter (Shimadzu Corporation, Kyoto, Japan) to evaluate surface/local spatial homogeneity and nanomechanics of the films. A total of 8 distinct locations were randomly selected across the sample surface to obtain a representative average while ensuring sufficient distance between indentations to avoid plastic zone overlap. The indentation tests consisted of loading to a maximum load of 10 mN at a speed of 1.4632 mN/s (on a 5 µm depth scale), a 5 s loading time at maximum load, and unloading at the same speed. Afterward, the indentation depth was continuously recorded for 5 s upon reaching the minimum load of 0.02 mN.

The bulk strength of composite films was evaluated via tensile testing using a Q800 dynamic mechanical analyzer (TA Instruments, DMA, New Castle, DE, USA). Rectangular specimens with dimensions of 20 mm × 3 mm (length × width) were tested by striped clamps. The tests were carried out in isothermal mode at 35 °C, with a force ramp of 3 N/min up to 18 N to cause fracture of the specimen. Stress–strain curves were derived from the recorded data.

## 3. Results and Discussion

### 3.1. Characterization of PVA/fLGAS Composite Films

**Figure 2:**
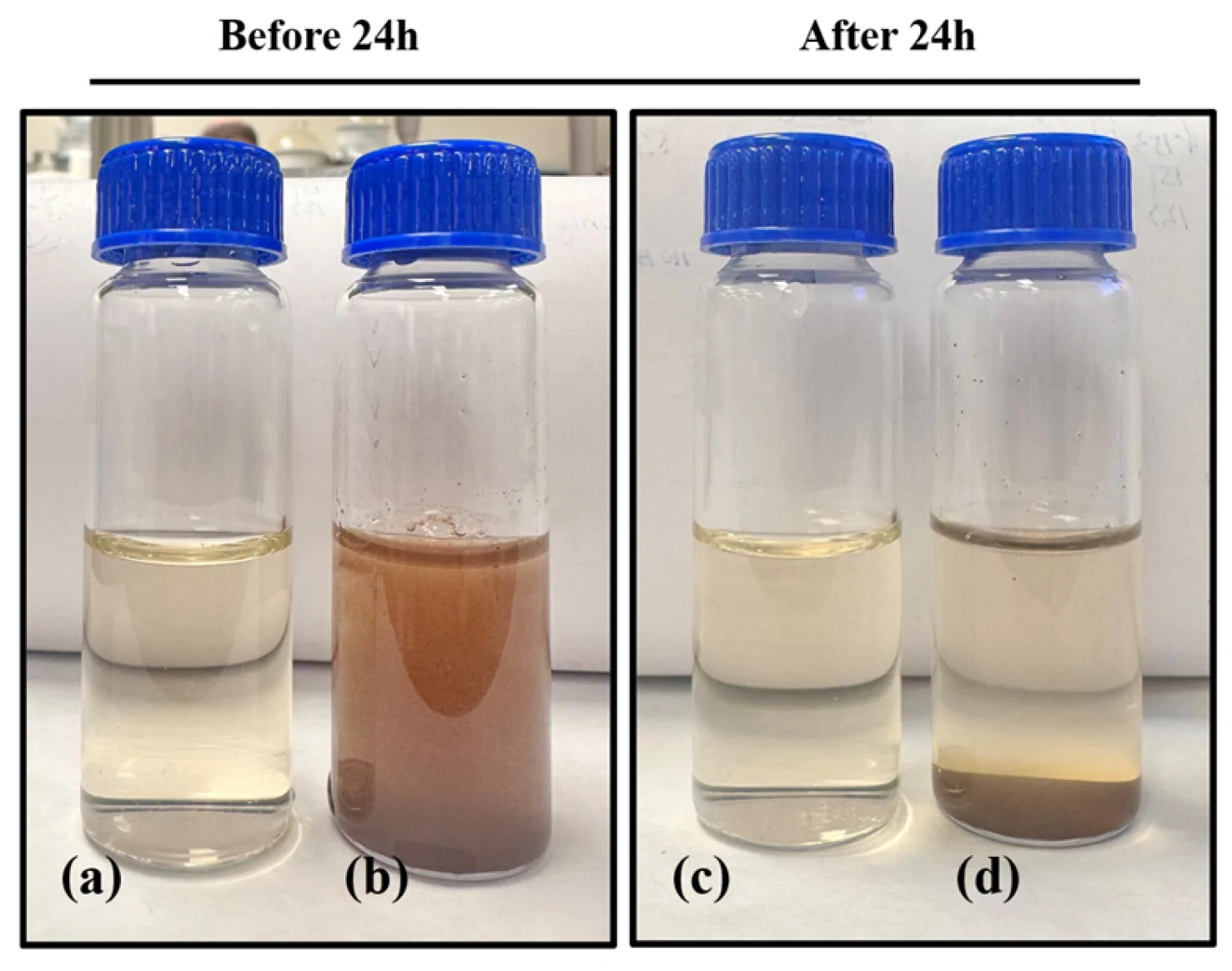
Aqueous suspensions (0.025 g/10 mL) of (**a**) fLGAS; (**b**) kLGAS; (**c**) fLGAS after 24 h; (**d**), and kLGAS after 24 h.

The aqueous dispersibility of fLGAS and kLGAS was first evaluated to assess the effect of enzymatic modification on lignin solubility. As shown in Figure 2a,b, clear differences were observed between the two lignin suspensions (25 mg lignin in 10 mL distilled water). The fLGAS suspension exhibited a lighter color and partial transparency, indicating improved dispersion in water, whereas the kLGAS suspension displayed a dark brown appearance characteristic of poorly dispersed lignin. After 24 h (Figure 2c,d), kLGAS underwent complete precipitation, while the fLGAS suspension remained stable without visible sedimentation. These results demonstrate that enzymatic modification significantly enhanced the aqueous dispersibility and stability of lignin.

The improved solubility and dispersion behavior of fLGAS can be attributed to the structural modifications induced by enzymatic treatment. In our research group, we demonstrated that enzymatic processes altered lignin side-chain configurations and increased the abundance of specific functional groups. The introduction of hydrophilic functionalities and disruption of hydrophobic domains contributed to enhanced lignin solubility, which is essential for achieving improved compatibility and uniform dispersion within the PVA matrix. The FTIR characterization of the functionalized lignin structure is shown in Figure S1. As shown in the SEM micrographs (Figure S2 in the Supplementary Information), all composite films display a smooth, dense, and homogeneous cross-sectional structure without visible micro-phase separation or large filler agglomeration. This uniform morphological feature directly proves that fLGAS was completely dissolved in the PVA matrix at the molecular level.

**Figure 3:**
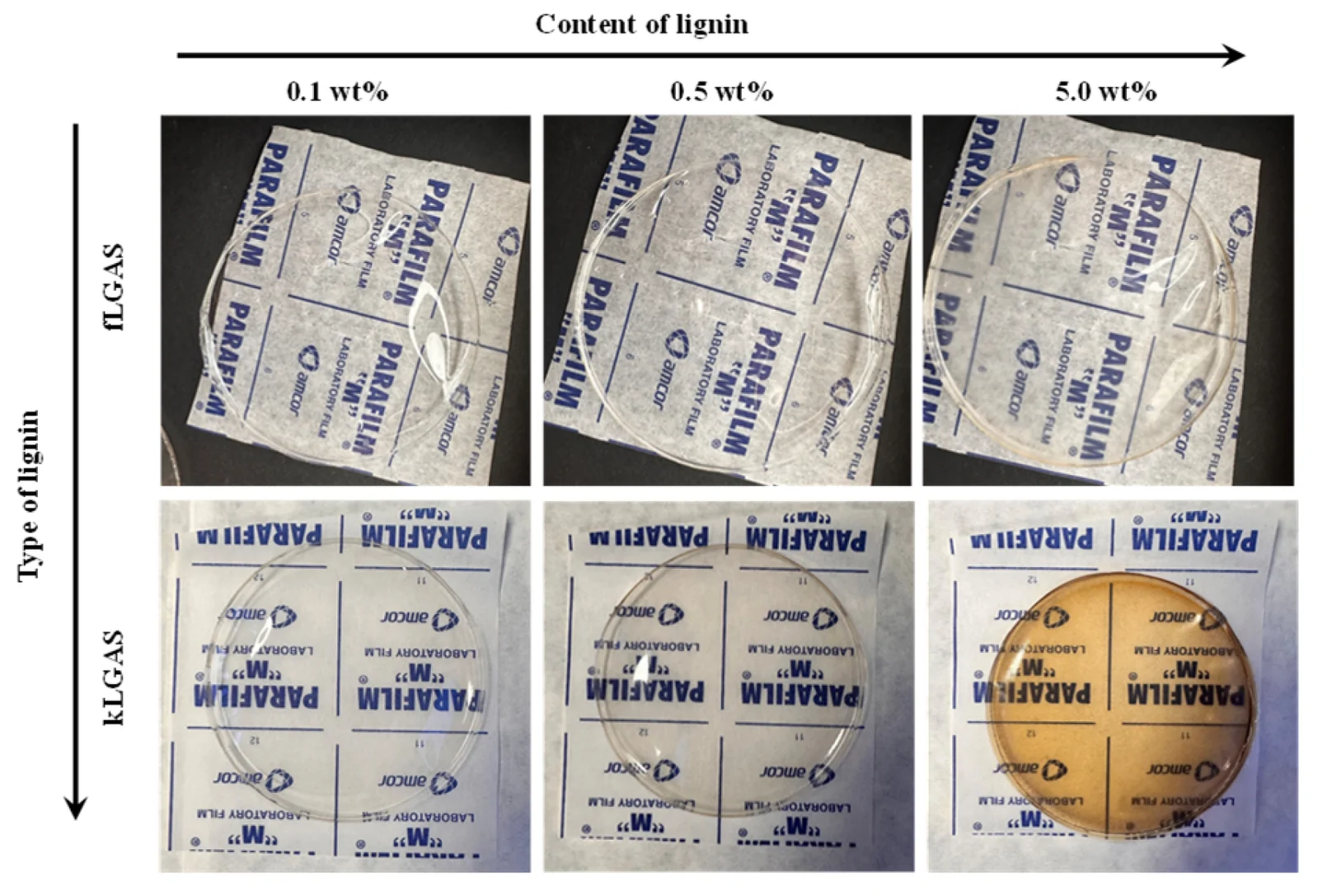
Macroscopic morphology of the composite films.

The composite films were prepared according to the procedure described in the Materials and Methods and subsequently characterized to evaluate their structural and properties. As shown in Figure 3, the films containing kLGAS exhibited noticeable differences in color compared with the other formulations. Furthermore, visible lignin aggregates were observed in the films containing 5 wt% lignin, particularly when comparing fLGAS and kLGAS containing samples with the same lignin loading. These differences can be attributed to the distinct solubility and dispersion behaviors of kLGAS and fLGAS within the PVA matrix. The improved dispersibility of fLGAS facilitates a more homogeneous distribution throughout the composite film, whereas the poor solubility of kLGAS promotes aggregation and heterogeneous film morphology.

#### 3.1.1. Fourier Transform Infrared Spectroscopy (FTIR)

**Figure 4:**
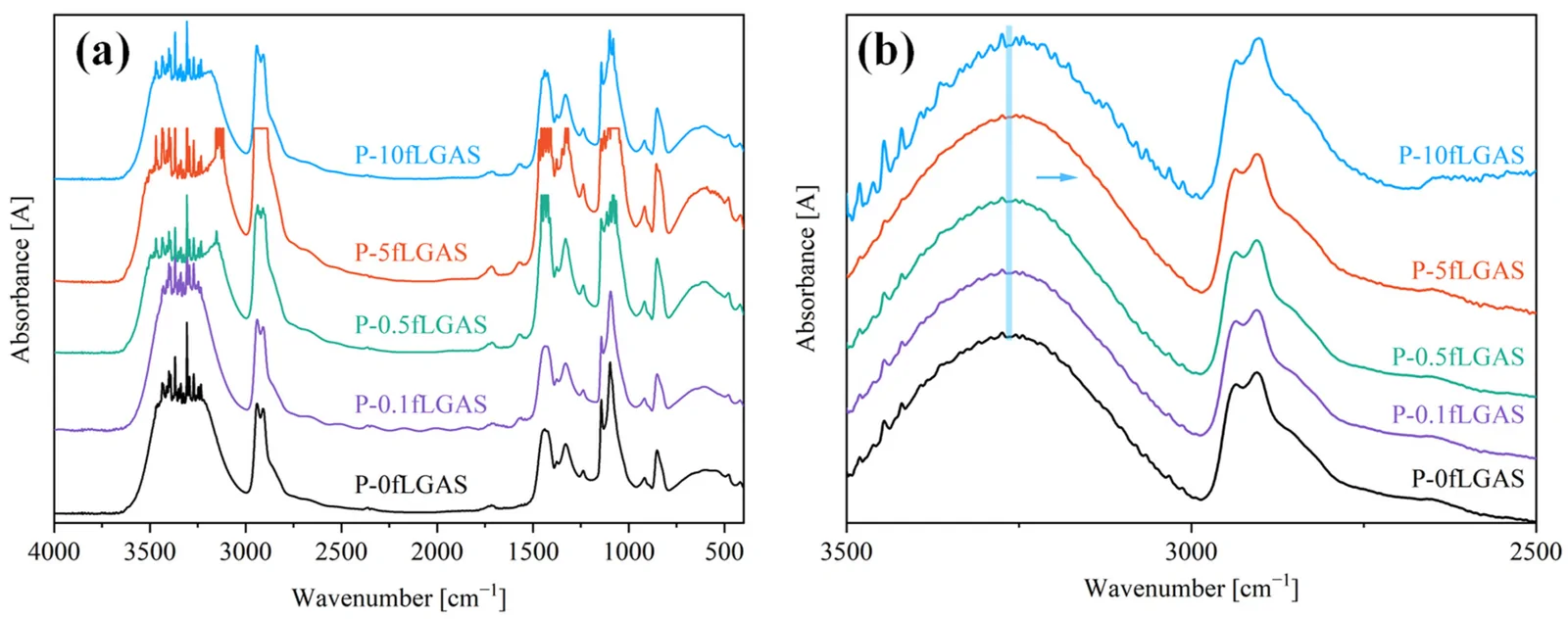
(**a**) Absorption FTIR spectra of PVA/fLGAS films; (**b**) ATR-FTIR spectra of PVA/fLGAS films. The blue vertical line indicates the peak position of P-0fLGAS, and the blue arrow denotes the peak shift toward lower wavenumbers with increasing fLGAS content.

As part of the characterization, the molecular features, and the effect of fLGAS content on the intramolecular and intermolecular interactions in composite films, of PVA/fLGAS films were characterized by FTIR and ATR-FTIR (Figure 4).

Pure PVA (P-0fLGAS) exhibits representative absorption peaks at 2940 cm^−1^ and 2909 cm^−1^ (asymmetric and symmetric stretching vibrations of C-H_2_), 1717 cm^−1^ (C=O vibration), 1433 cm^−1^ and 1323 cm^−1^ (asymmetric and symmetric bending vibrations of C-H), and 1105 cm^−1^ (C-O vibration), as shown in Figure 4a [16]. The composite films display an additional absorption peak at 1572 cm^−1^, corresponding to the stretching vibration of the C=C skeleton in the benzene ring from lignin in comparison with pure PVA. Nevertheless, other differences were not observed between the numerous composite films [17], which may be attributed to the limited changes in the chemical environment induced by fLGAS incorporation, the overlapping characteristic absorption bands of PVA and fLGAS that hindered the detection of subtle molecular variations, and the excessive film thickness (0.045–0.060 mm) that may have reduced the sensitivity of the analysis. Additionally, the high thickness of the composite films may have caused absorbance saturation in regions with intense molecular vibrations, resulting in plateau-shaped peaks where the maximum absorbance exceeded the detector’s linear response range. This effect can mask the actual peak intensity and position, limiting the identification of subtle molecular changes. Therefore, ATR-FTIR analysis was employed to provide a more accurate evaluation of the molecular interactions within the composite films. Figure 4b shows a redshift of the O-H band toward lower wavenumbers as lignin content increases, with peak positions at 3268 cm^−1^ for pure PVA, 3255 cm^−1^ for 5 wt% lignin, and 3248 cm^−1^ for 10 wt% lignin. This shift could theoretically result from either a change in the tacticity of PVA or the formation of hydrogen bonds. Given that the blending of PVA and fLGAS is a purely physical mixing process, the possibility of altering the polymer’s tacticity could be safely ruled out. Therefore, the observed shift can be definitively attributed to the formation of hydrogen bonds between PVA and fLGAS [18].

#### 3.1.2. X-Ray Diffraction (XRD)

**Figure 5:**
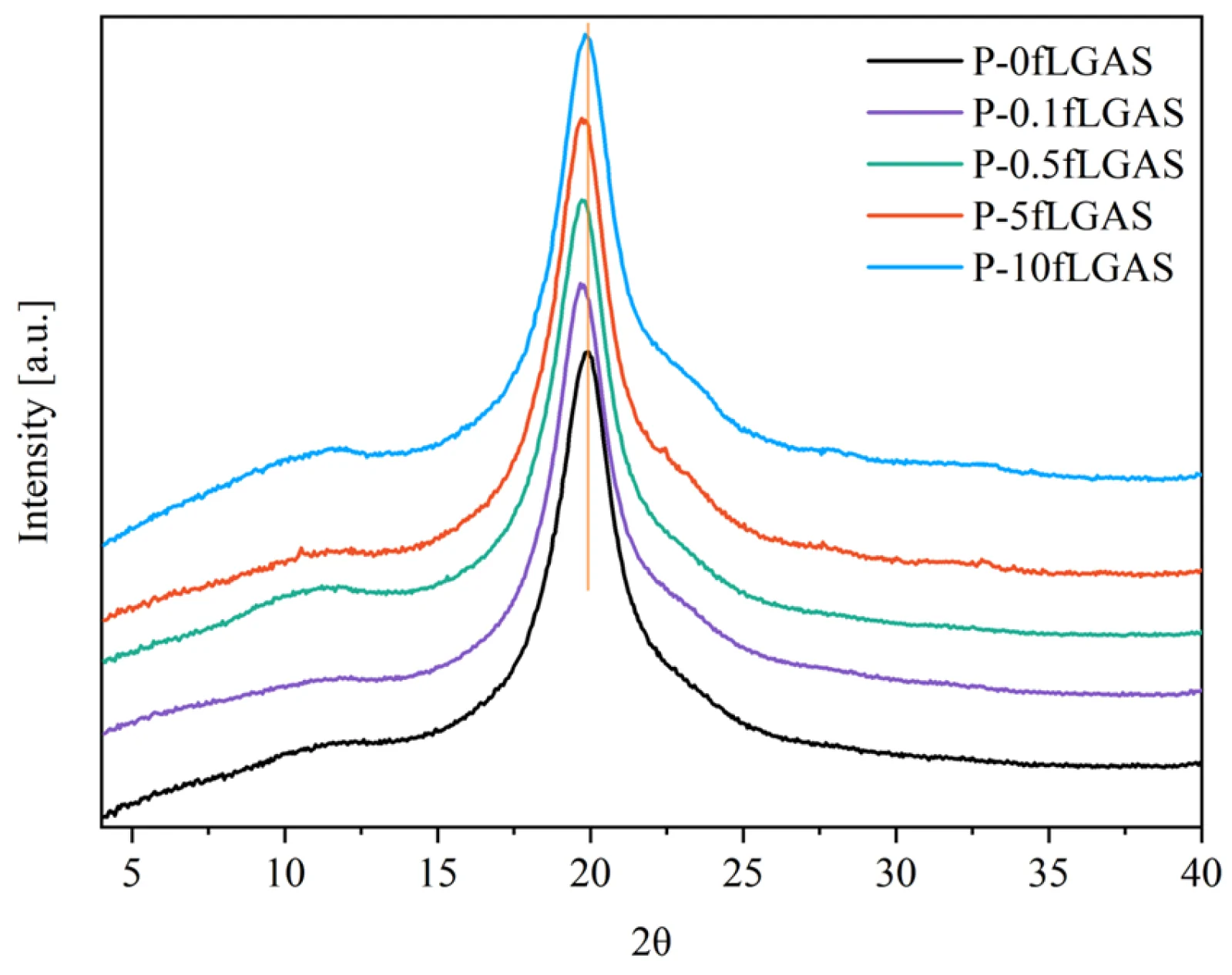
XRD spectra of PVA/fLGAS films. The orange vertical line indicates the peak position of P-0fLGAS.

Figure 5 presents the XRD patterns for PVA/fLGAS composite films, and the peak positions and d-spacing values are summarized in Table 2. Pure PVA displays a prominent diffraction peak at 2*θ* = 19.9°, together with two less intense distinct peaks at 2*θ* = 23.8° [19], which are characteristic of its semicrystalline structure. In contrast, fLGAS does not display well-defined diffraction peaks because its structure is predominantly amorphous, contributing instead to a broad diffuse halo rather than distinct crystalline reflections. With incorporating increasing amounts of lignin, the main diffraction peak of PVA shifts slightly toward lower diffraction angles, from 19.94° to 19.78°. This shift indicates a progressive expansion in the interplanar spacing (d-spacing), reflecting crystalline lattice distortion within the PVA matrix according to Bragg’s Law. This effect can mainly be attributed to the formation of strong intermolecular hydrogen bonds between the hydroxyl groups of lignin and PVA, which partially disrupts the packing of PVA chains and is consistent with the FTIR results [20].

Upon the incorporation of lignin, the amorphous region of the composite films (2*θ* = 10–25°) increases compared with pure PVA, reflecting the increasing contribution of the predominantly amorphous lignin phase. Nevertheless, even for P-10fLGAS, which contains the highest lignin content, the crystallization behavior of PVA is not significantly suppressed. As the fLGAS content increases, however, the broad amorphous halo increasingly overlaps with the PVA crystalline reflections, reducing their resolution and making reliable peak deconvolution difficult.

Regarding the interplanar spacing, compared with pure PVA (19.94°, d = 4.45 Å), all composite films exhibited a clear shift in the main diffraction peak toward lower 2*θ* (19.67–19.78°), corresponding to an increase in interplanar distance (d-spacing, 4.49 Å–4.51 Å). This lattice expansion demonstrates that the introduction of fLGAS disrupted the tight crystal packing of PVA chains via intermolecular hydrogen bonding. Within the composite series, increasing fLGAS loading led to a minor reduction in d-spacing (from 4.51 Å at 0.1 wt% to 4.49 Å at 10 wt%), which is attributed to spatial confinement and local self-aggregation of fLGAS at higher concentrations.

### 3.2. Properties

#### 3.2.1. Thermal Properties

**Figure 6:**
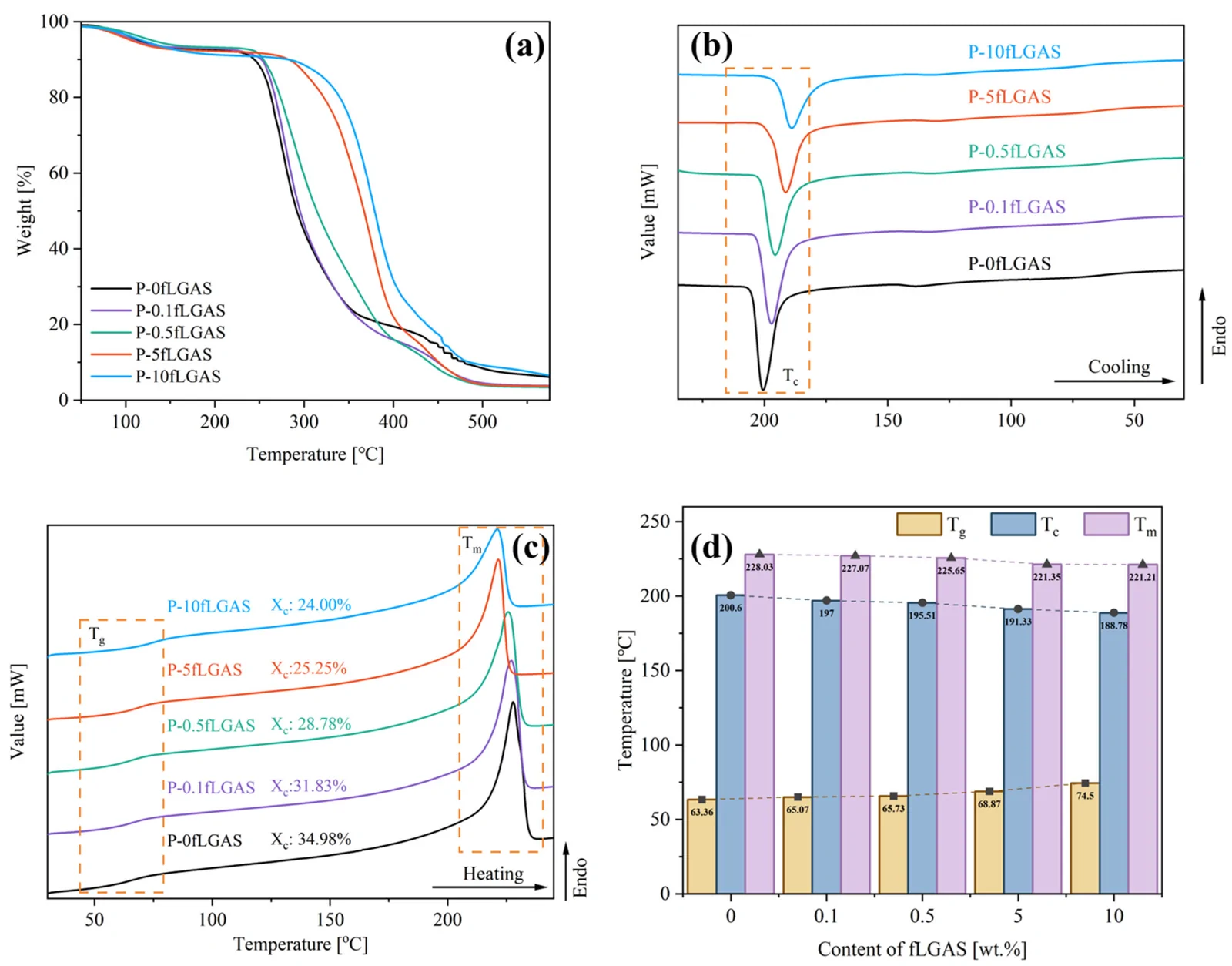
(**a**) TGA curve; (**b**) DSC cooling curve; (**c**) DSC heating curve; (**d**) statistics and analysis of PVA/fLGAS films (symbols represent the numerical values of the columns, and the dashed line connects these values).

Figure 6 illustrates the studies of the thermal stability of the PVA/fLGAS films carried out by TGA and DSC. The TGA curves exhibit three distinct stages of weight loss (Figure 6a): (1) evaporation of residual solvent between 50 and 150 °C, (2) PVA decomposition and fLGAS degradation between 200 and 400 °C, and (3) char formation above 400 °C across all the samples. It is clear that the formation of hydrogen bonds between PVA and fLGAS enhances the thermal stability of the films as fLGAS content increases. Specifically, the thermogravimetric curves reveal a significant rightward shift in the main degradation stage, with the onset decomposition temperature increasing progressively from approximately 250 °C for pure PVA to nearly 300 °C for the 10 wt% blend. This pronounced delay could confirm that the dense intermolecular interaction network effectively restricts the thermal motion of the PVA chains. These findings align with results reported by Rahmani, A. [21], who demonstrated that crosslinking in PVA-based films improves thermal stability.

On the other hand, DSC analysis was conducted to evaluate the films’ thermal behavior under different treatments. As shown in the cooling curves from the molten state (Figure 6b), a clear shift toward lower crystallization temperatures (Tc) occurs as the fLGAS content decreases. This trend is further supported by the subsequent heating cycle (Figure 6c), where samples with lower fLGAS concentrations exhibit reduced melting points (Tm). This behavior is directly linked to a lower degree of crystallinity, as inferred from the reduced areas of the endothermic and exothermic peaks. In greater depth, the glass transition temperature (Tg), melting temperature (Tm), and crystallization temperature (Tc) were estimated according to the DSC curve (Figure 6d). The peaks placed at approximately 200 °C observed during the exothermic processes correspond to the crystallization of the composite films. Tc shifts toward lower temperatures with increasing fLGAS content, attributed to interactions between PVA and fLGAS. These interactions disrupt the regular packing of PVA chains, restrict segmental mobility, and delay chain folding, thereby suppressing the overall crystallization rate [22]. Similarly, Tm shifts downward from 228.03 °C to 221.21 °C, following the same mechanism as Tc [23]. The endothermic curve (Figure 6c) shows both Tm and a step-change corresponding to Tg. Unlike Tc and Tm, Tg increases with higher fLGAS content (rising from 63.36 °C to 74.5 °C). This rise is driven by restricted chain mobility upon fLGAS incorporation, which hinders the relaxation of amorphous chains and delays the transition from the glassy to the rubbery state [24].

Additionally, the degree of crystallinity (Xc) of each composite was determined from the second heating scan (Figure 6c). Specifically, the experimental melting enthalpy ($∆H_{m}$) obtained during the heating process was used to calculate Xc according to Equation (2) [25]:

(2) $$X_{c}=\frac{{∆H}_{m}}{{∆H}_{m}^{100\%}\times \omega }$$

The melting enthalpy of an ideal crystal of $∆H_{m}^{100\%}$ is 138.6 $J\cdot g^{-1}$ [26]. ω represents the concentration of PVA. The calculated Xc values for P-0fLGAS, P-0.1fLGAS, P-0.5fLGAS, P-5fLGAS, and P-10fLGAS were 34.98%, 31.83%, 28.78%, 25.25%, and 24.00%, respectively. The progressive reduction in PVA crystallinity upon fLGAS addition indirectly confirms the formation of intermolecular hydrogen bonds, which disrupt regular polymer chain arrangement (Figure 6d).

To quantitatively elucidate the hydrogen-bonding network, peak deconvolution and hydrogen bond fraction calculations [27] were performed on the FTIR-OH stretching band, with the resulting parameters and crystallinity Xc summarized in Table 3.

As fLGAS content increases, a clear, monotonic shift in the hydrogen-bonding topology is observed. Specifically, the fraction of OH⋯ether O interactions (where “⋯” denotes hydrogen bonding) systematically increases from 61.77% in neat PVA to 73.35% in P-10fLGAS, whereas the cyclic OH fraction decreases from 24.21% to 8.46%. This evolution directly demonstrates that the abundant ether oxygen and methoxy acceptors on fLGAS effectively disrupt the intrinsic cyclic H-bonded clusters of PVA, converting them into a dominant, highly interconnected interfacial H-bonded network. Concurrently, the overall FTIR absorption peak exhibits a continuous red-shift from 3268 to 3248 cm^−1^, verifying the macroscopic strengthening of the physically crosslinked network. This high-density interfacial crosslinking, together with the steric hindrance imposed by the rigid fLGAS domains, limits PVA chain mobility. Consequently, the crystallinity Xc demonstrates a steady decline from 34.98% to 24.00%, as chain alignment into ordered crystalline lattices is substantially hindered.

#### 3.2.2. Swelling Properties and Water Contact Angle (WCA)

**Figure 7:**
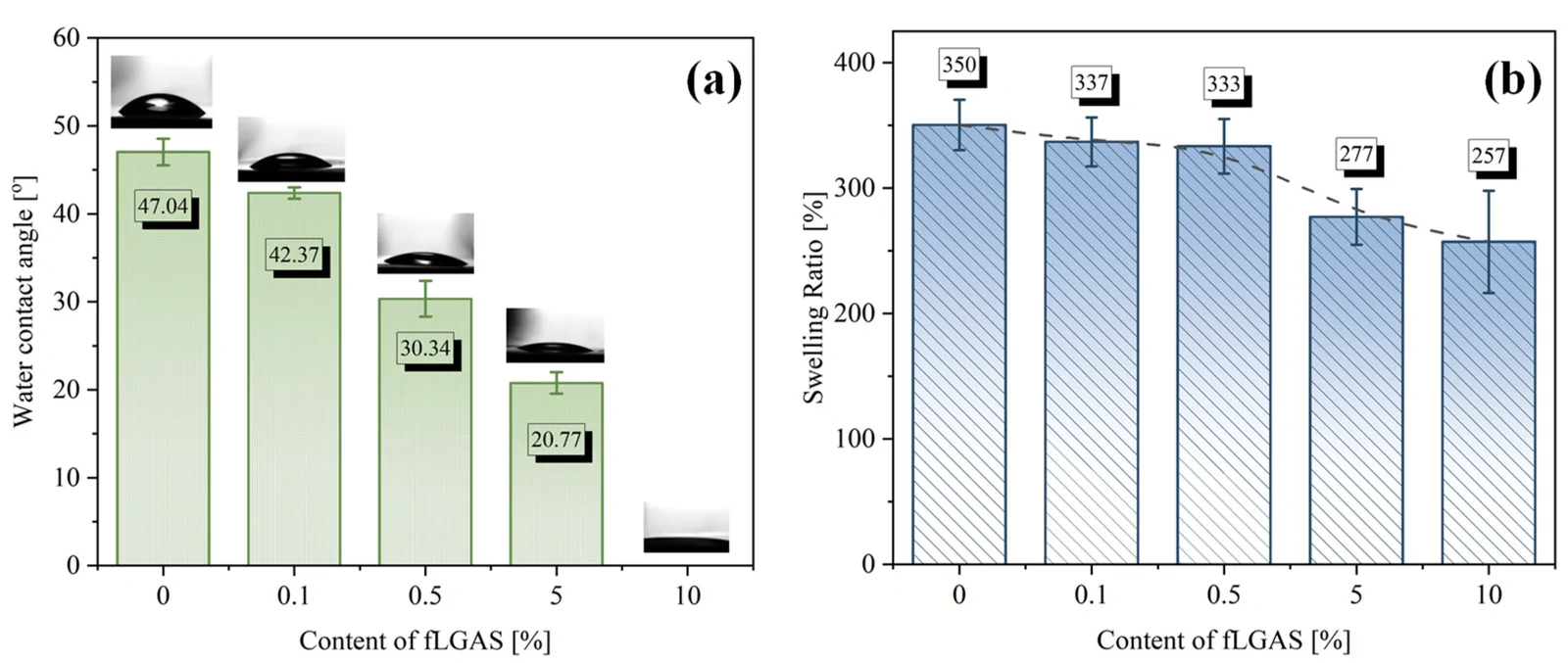
(**a**) Water contact angle and (**b**) swelling ratio (2nd h) of PVA/fLGAS films (data are expressed as the mean ± standard deviation (SD) of three independent experiments). The markers above the bars represent the values of each column, and the dashed line represents a B-spline curve connecting these data points.

The swelling properties and WCA were also studied for all the prepared samples. Previous studies with unfunctionalized lignin reported that adding lignin increases the WCA [28,29]. However, the present study shows the opposite trend, indicating enhanced hydrophilicity of composite films with fLGAS incorporation. As shown in Figure 7a, the WCA of pure PVA is 47.04°, decreasing to 20.77° at 5 wt% fLGAS. For P-10fLGAS, water is absorbed almost instantaneously upon contact, making it impossible to measure a contact angle. This observation aligns with the higher solubility of the fLGAS solution and the FTIR spectra, which show increased O-H groups contributing to greater hydrophilicity. Although hydrogen bonding consumes some -OH groups, the addition of lignin introduces other hydrophilic functional groups, leading to a corresponding decrease in the water contact angle. Additionally, the decreased crystallinity increases the fraction of amorphous domains; the higher segmental freedom in these regions facilitates rapid water penetration and wetting.

On the other hand, Figure 7b shows that the actual trend of the swelling ratio deviates significantly from the anticipated tendency inferred from the WCA results. With increasing fLGAS content, the swelling ratio exhibits a slight downward trend, particularly at higher concentrations (P-5fLGAS and P-10fLGAS). Nevertheless, the overall swelling capacity remains within the 270–350% range. This is primarily attributed to the inherently lower water absorption capacity of lignin compared to PVA [30]. Secondly, the hydrogen bonding between PVA and fLGAS acts as a physical crosslink, which restricts the expansion of the polymer chains [31]. Consequently, although the presence of more O-H groups enhances the surface energy for water absorption, the reinforced network resists excessive water uptake.

#### 3.2.3. Mechanical Properties

To establish a comprehensive mechanical profile, dynamic mechanical analysis and nanoindentation were combined to evaluate bulk viscoelasticity and local/surface mechanics, respectively. While DMA captures the macroscopic bulk response, nanoindentation mapping provides complementary sub-micron resolution, confirming uniform fLGAS dispersion without the localized phase separation that bulk measurements typically average out. Additionally, nanoindentation isolates surface-specific elasticity and localized creep behavior under micro-Newton loads, effectively bridging bulk thermomechanical performance with surface contact mechanics.

##### Nanoindentation

**Figure 8:**
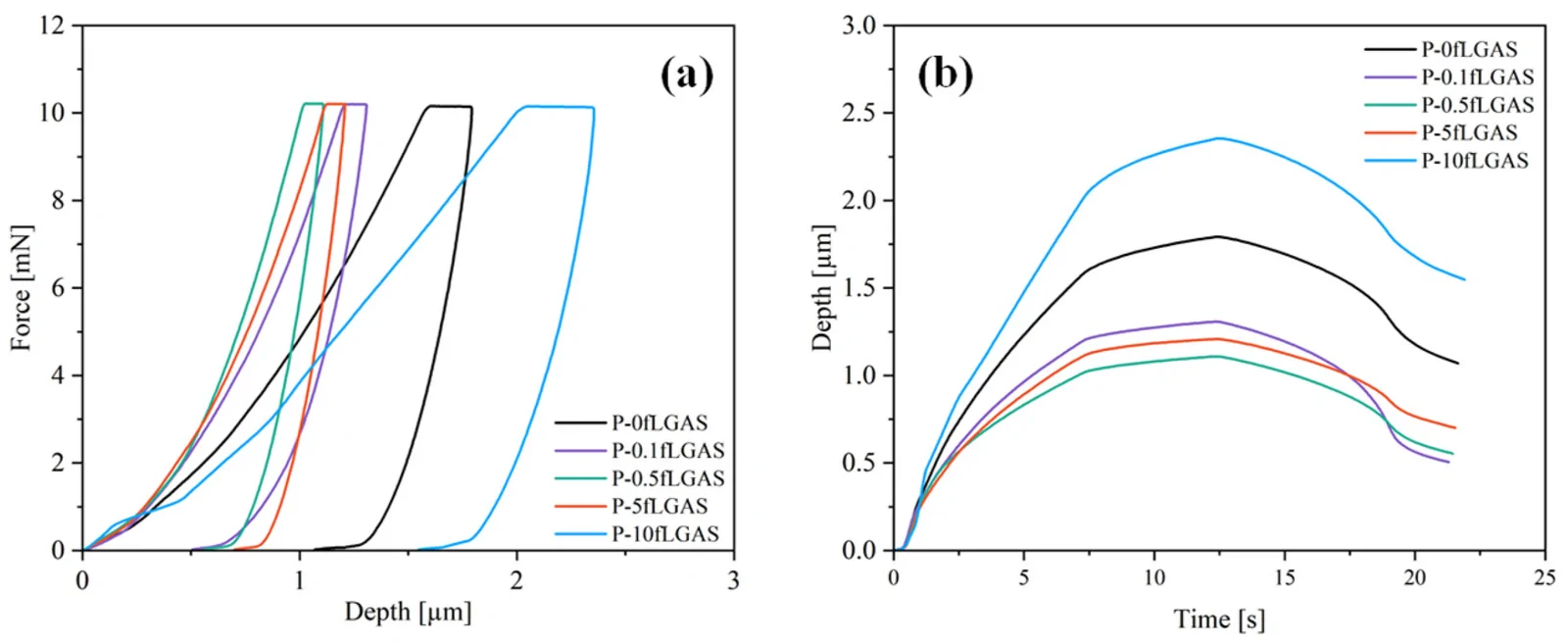
(**a**) Load-indentation depth curves and (**b**) indenter depth dependence on time.

Figure 8 illustrates the loading, holding, and unloading stages during depth-sensing nanoindentation experiments for the PVA/fLGAS films. As shown in the load–indentation depth curves (Figure 8a), the maximum penetration depth and curve shapes vary significantly across compositions. The P-0.5fLGAS sample exhibits the highest resistance to penetration, resulting in the smallest maximum depth and minimal creep deformation during the holding phase. This enhanced hardness due to strong intermolecular hydrogen bonding between PVA and optimal fLGAS contents, which restricts polymer chain mobility. Conversely, the pristine film (P-0fLGAS) and the highest-loaded sample (P-10fLGAS) exhibit substantially deeper indenter penetration and greater permanent plastic deformation after unloading. For P-10fLGAS, this reduction in hardness is attributed to two main factors: (i) reduced PVA crystallinity, which weakens the semi-crystalline network, and (ii) filler aggregation at high loadings, where excess fLGAS acts as structural defects that disrupt matrix continuity and promote plastic flow.

Figure 8b displays the variation in indenter depth as a function of time for the PVA/fLGAS samples. The maximum depth reached at the end of the loading period, at approximately 12.5 s, is highly dependent on the composition. Notably, the most remarkable difference is noted between the P-0fLGAS and P-10fLGAS. Meanwhile, the remaining three samples reach comparable depths, and significantly reduce the maximum depth from 1.8 μm (P-0fLGAS) to approximately 1.1–1.3 μm, consistent with the previous discussion. The unloading process is primarily dominated by the viscoelastic recovery of the different films. Accordingly, the films do not exhibit purely elastic behavior; their permanent deformation is evidenced by the depth dependence on time during unloading. The P-0.1fLGAS, P-0.5fLGAS and P-5fLGAS exhibit much lower permanent deformation compared to the P-0fLGAS and P-10fLGAS. These results indicate a strong reinforcing effect, where the hydrogen bonding enhances the resistance to indentation and improves both the stiffness and the elastic recovery capability at lower loadings of fLGAS. This trend is in good agreement with previous nanoindentation studies on PVA-based composite films [32].

DSI also provided the creep response of the films during the holding stage, where the load was maintained constant between the loading and unloading cycles. Under a constant load, the indentation depth increases from t = 7.5 s to t = 12.5 s. An indentation pseudo-compliance was introduced (Equations (3) and (4)) as a basis for comparing viscoelastic behavior across different samples. Here, strain is represented by the relative depth increment, and stress is substituted with the Martens hardness, the latter corresponding to the mean contact pressure applied by the indenter.

(3) $$h\left(t_{cr}\right)=h_{0}+∆h(1-\exp(-{{(t}_{cr}/\tau )))}^{\beta }$$

(4) $$J_{it}^{*}\left(t_{cr}\right)=\frac{(h\left(t_{cr}\right)-h_{0})/h_{0}}{\mathrm{HMs}}$$

where t_cr_ represents the time elapsed since the onset of the holding period; h_0_ is the penetration depth at t_cr_ = 0; ∆h denotes the extrapolated displacement of the indenter for an infinite holding time; *τ* is the effective retardation time; and *β* is the stretching exponent (0 < *β* ≤ 1).

**Figure 9:**
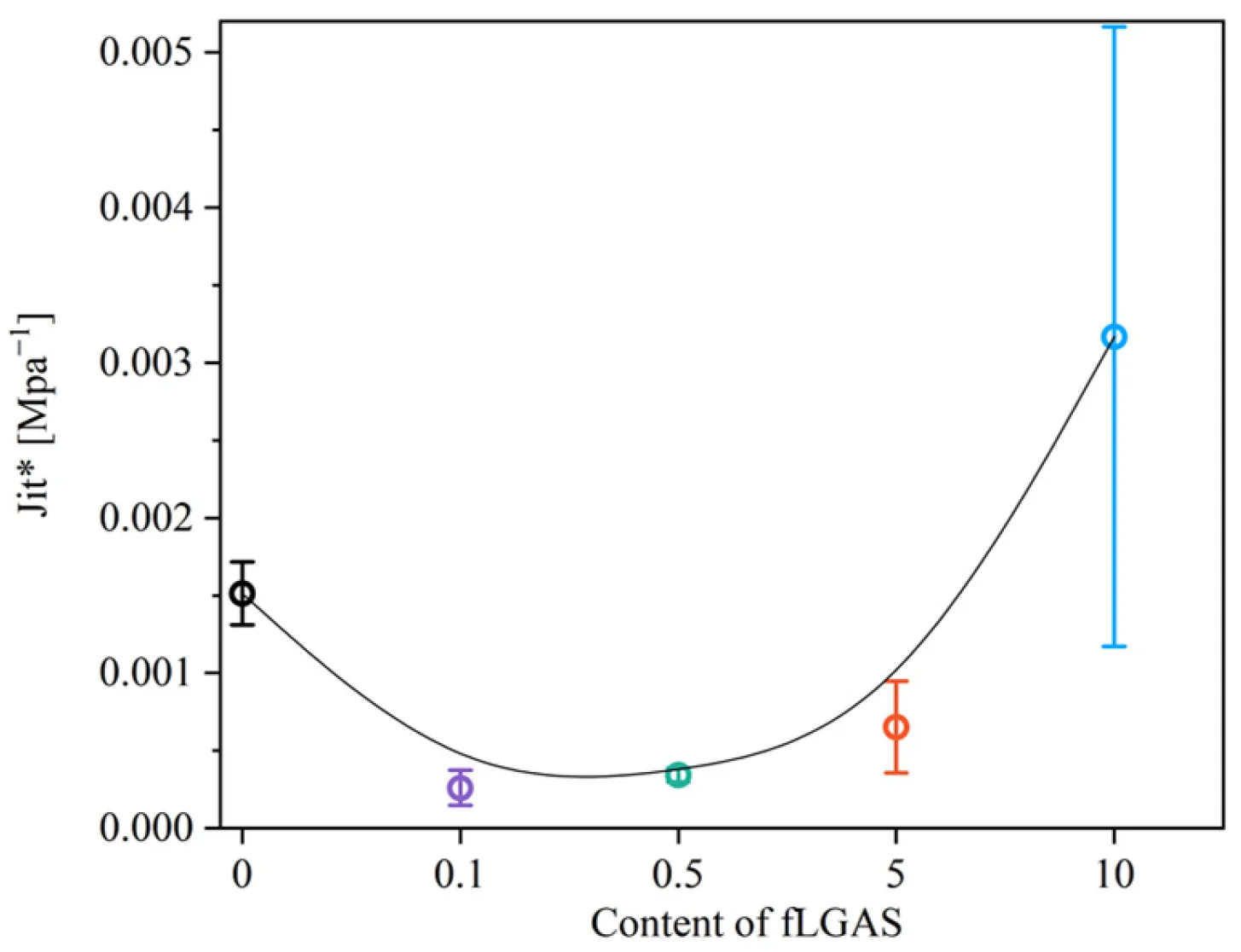
Variation in asymptotic value of the indentation pseudo-compliance, Jit*, with global content of fLGAS (data are expressed as the mean ± standard deviation (SD) of eight independent experiments).

As shown in Figure 9, the P-10fLGAS film demonstrates the highest creep compliance during the holding phase. This indicates that the film undergoes significant time-dependent plastic deformation under sustained load. This behavior aligns with the reduced hardness observed in Figure 8a. It is driven by the softening effect of excess fLGAS, which makes the film more compliant [33]. While a more compliant film offers superior surface conformability, its heightened plasticity and compromised elastic recovery compromise structural stability during handling. Consequently, films with lower fLGAS loadings (e.g., P-0.5fLGAS) balance mechanical integrity with flexibility.

##### Dynamic Mechanical Analysis (DMA)

**Figure 10:**
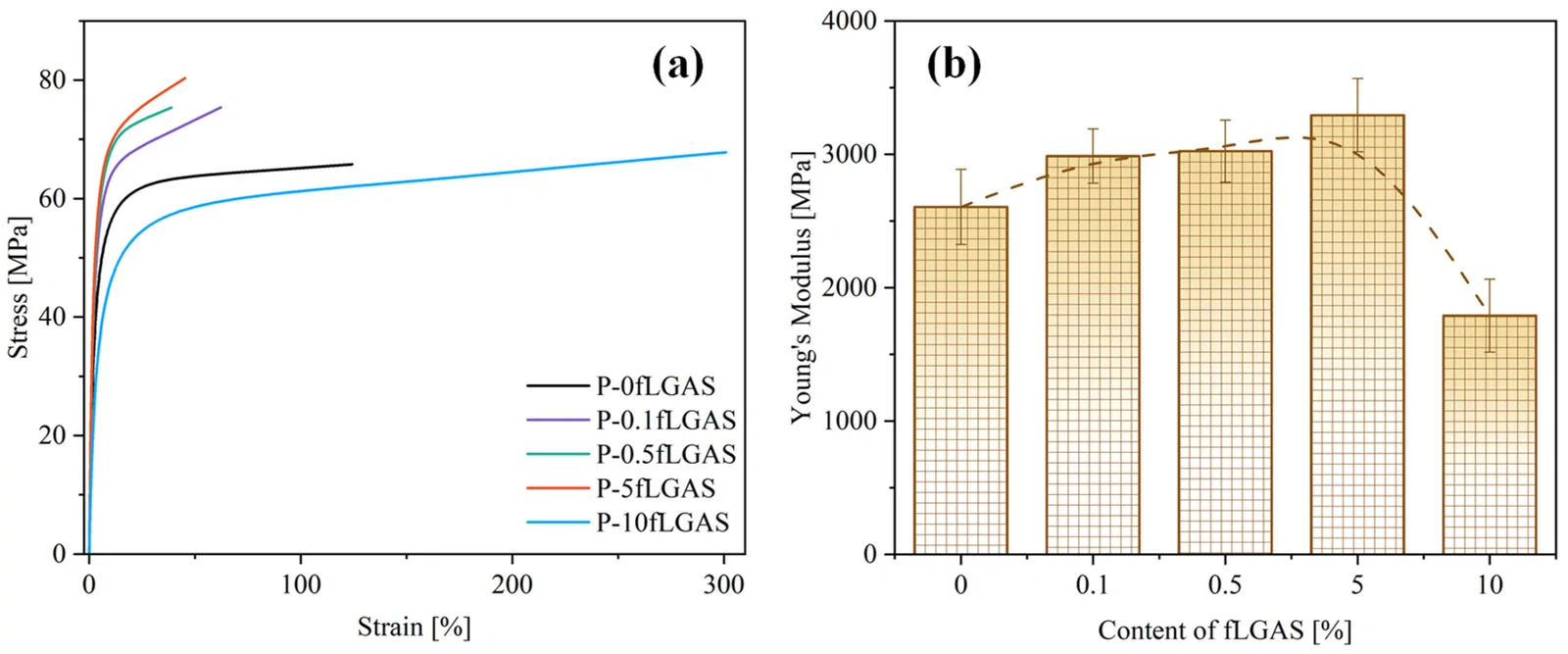
(**a**) Tensile test; (**b**) Young’s modulus of PVA/fLGAS films (the dashed line represents a B-spline curve connecting the mean values).

Tensile DMA was performed at 35 °C to evaluate the mechanical properties of the PVA/fLGAS films (Figure 10a,b). As the fLGAS loading increased up to 5 wt%, Young’s modulus progressively rose, peaking at 3293.2 MPa. This initial enhancement is driven by extensive intermolecular hydrogen bonding, where fLGAS acts as a physical crosslinker that restricts PVA chain mobility and reinforces the amorphous matrix. However, the overall mechanical performance reflects a competing balance between fLGAS reinforcement and the reduction in PVA crystallinity (Xc). At higher loadings (P-10fLASG), the loss of crystalline domains—which normally act as load-bearing nodes in semi-crystalline PVA—dominates over the crosslinking effect. Combined with fLGAS agglomeration, which creates structural defects and stress concentration sites, this reduction in crystallinity causes a sharp decline in both the Young’s modulus and yield stress.

The general trend aligns with the nanoindentation results; however, it also reveals distinct characteristics not captured by the former. Firstly, a significant discrepancy in modulus values was observed, where nanoindentation results were approximately 2 times higher than those from DMA. These pronounced differences are primarily attributed to the elevated testing temperature [34]. Due to the moisture–temperature coupling effect in PVA, the film undergoes a dramatic glass transition as humidity and temperature increase, leading to a sharp decline in the storage modulus [35]. Consequently, the DMA measurement at 35 °C captured the post-transition rubbery state, whereas the nanoindentation at 20 °C reflected the rigid glassy state. Moreover, the difference in modulus values is partially driven by the distinct mechanisms of the respective testing methods. Specifically, dynamic nanoindentation assesses the localized viscoelastic response through compressive stress field on surface, whereas DMA in tensile mode evaluates the bulk properties under uniaxial oscillatory stretching. These fundamentally different stress states, combined with the varying testing position, inevitably lead to the observed offset in absolute modulus values.

Another difference concerns the optimal mechanical performance of the films. Based on DMA measurements, P-5fLGAS exhibits superior mechanical properties; however, according to nanoindentation testing, P-0.5fLGAS demonstrates the highest performance. These nanoindentation results do not contradict the DMA findings; rather, they provide a perfect complementarity. These data demonstrate that lignin, acting as an amorphous agent, maximizes localized microscopic stiffness and plastic deformation resistance at ultra-low concentrations (0.1–0.5%). At 5% loading, while the material achieves optimal crystallinity and macroscopic mechanical performance, its intrinsic amorphous flexibility begins to manifest at the micro-scale, leading to a reduction in micro-hardness. Finally, the sharp decline at 10% loading consistently substantiates the catastrophic impact of severe agglomeration on multi-scale mechanical properties.

Table 4 presents more detailed DMA calculation results. As the fLGAS loading increased, the Young’s modulus, yield stress, and yield strain of the composite films exhibited a general “rise-and-fall” trend, culminating in a severe reduction at a loading of 10 wt% (P-10fLGAS). Specifically, the incorporation of moderate amounts of fLGAS (0.1 to 5 wt%) successfully reinforced the polymer matrix. The yield stress peaked at a low loading of 0.1 wt% (12.2 ± 3.46 MPa), representing a substantial increase compared to the neat PVA film (P-0 fLGAS, 7.73 ± 0.68 MPa). This enhancement can be attributed to the formation of hydrogen bonding between the well-dispersed fLGAS and the PVA matrix. However, further increasing the fLGAS content to 10 wt% led to a decline in all mechanical metrics; the Young’s modulus dropped to 1790.2 ± 273 MPa, and the yield stress and strain plummeted to 4.23 ± 1.98 MPa and 0.25 ± 0.13%, respectively, even falling below the corresponding values for neat PVA. This sudden mechanical degradation is likely associated with the reduction in crystallinity induced by the increased hydrogen bonding introduced at higher fLGAS concentrations, which disrupts the regular packing of the polymer chains and compromises the integrity of the polymer network.

**Figure 11:**
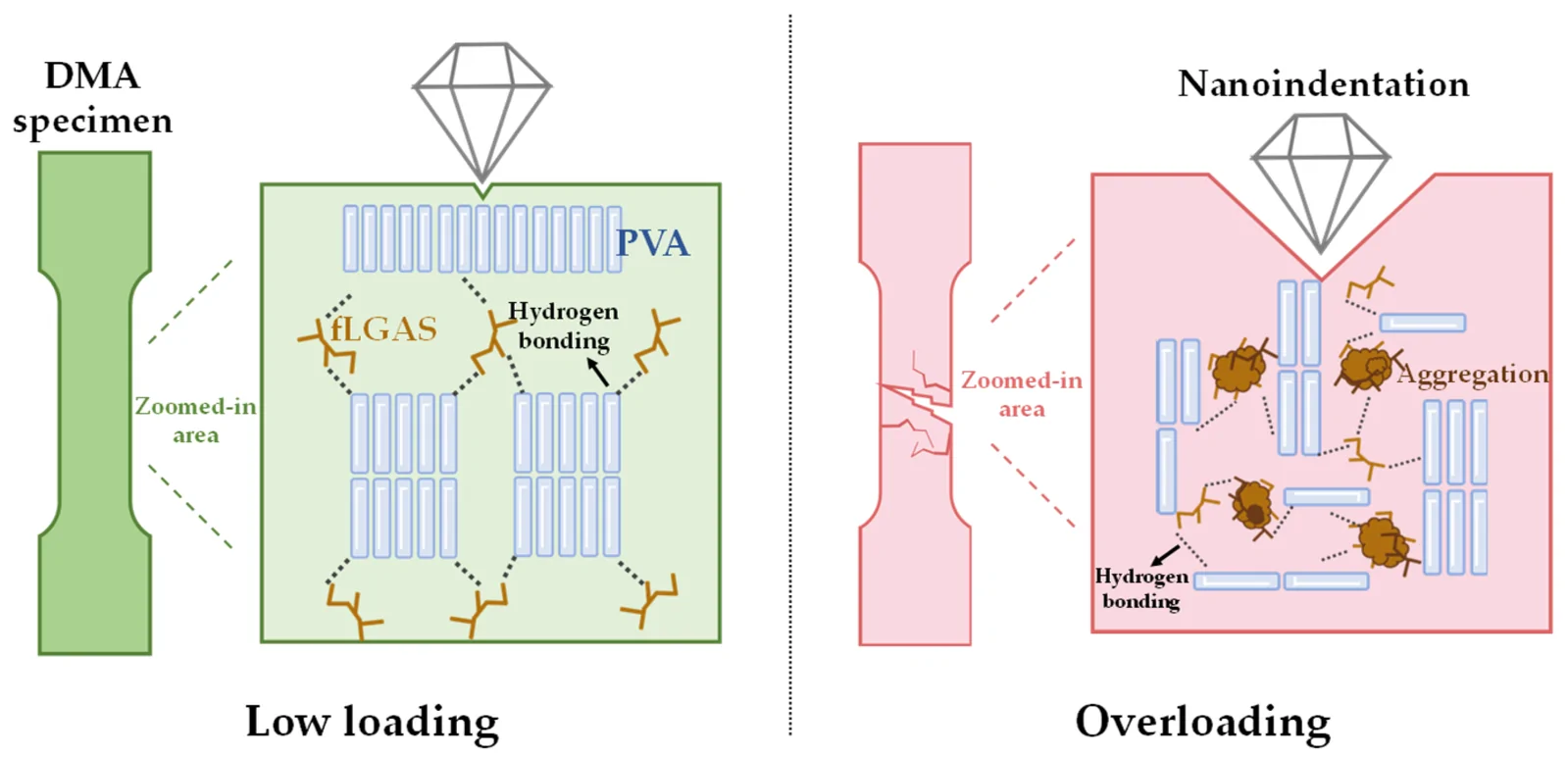
Schematic illustration of the molecular competitive interaction model.

As shown in Figure 11, based on the observed mechanical and structural variations, fLGAS concentration acts as a thermodynamic lever that controls the balance between two competing interactions: self-hydrogen bonding between PVA chains (PVA–PVA) and intermolecular hydrogen bonding between fLGAS and PVA (fLGAS–PVA). Pure PVA relies on strong self-interactions to form crystalline domains, while enzymatically modified fLGAS provides abundant hydroxyl groups that compete for PVA binding. Systematically tuning the fLGAS loading shifts the equilibrium across three distinct regimes: (1) Low Loading Regime: Dispersed fLGAS molecules bind locally to PVA chains through intermolecular hydrogen bonds without disrupting their long-range ordering. PVA chains retain enough mobility to pack tightly into crystalline domains, allowing high matrix crystallinity and strong local interfacial bonding to coexist and enhance mechanical properties. (2) Optimal Threshold: The density of intermolecular fLGAS–PVA hydrogen bonds reach an optimal saturation point. Interfacial adhesion between the lignin domains and the PVA matrix is maximized, ensuring efficient stress transfer without causing macroscopic aggregation. (3) High Loading Regime: Excessive fLGAS introduces severe steric hindrance and over-disrupts PVA self-hydrogen bonding. Surplus fLGAS molecules physically intercalate between PVA chains, blocking PVA–PVA alignment and suppressing crystallization, which directly accounts for the reduced crystallinity in the XRD patterns.

To evaluate mechanical property, the Young’s modulus of PVA/fLGAS (3293 MPa) was compared with other biopolymer systems (Table 5). The substantial enhancement over unmodified PVA/lignin (252 MPa) confirms efficient stress transfer via robust hydrogen-bonding networks.

Taken together, these mechanical findings highlight the distinct advantages of the present strategy. First, this approach effectively overcomes the long-standing “lignin weakening effect” commonly encountered in polymer engineering, where incorporating technical or unmodified waste lignin into PVA typically induces phase separation, stress concentration, and a drastic loss in tensile strength, even at low loadings. In contrast, our laccase-functionalized lignin (fLGAS) actively reinforces the matrix up to 5 wt% without property degradation. Second, this substantial reinforcement (~25% increase in tensile strength) is achieved through a chemical-free, green processing route that avoids toxic synthetic crosslinkers (e.g., glutaraldehyde, epichlorohydrin) or secondary coupling agents, relying entirely on robust intermolecular hydrogen bonding between fLGAS and PVA—a notable achievement for uncrosslinked bio-composites. Finally, this strength enhancement coincides with a 5 wt% reduction in synthetic PVA consumption, successfully fulfilling the dual objective of industrial biowaste valorization and petroleum-based polymer replacement without sacrificing matrix integrity.

The composite films demonstrate a two-phase behavior, wherein low loadings (0.1–0.5 wt%) provide baseline nanofiller reinforcement, whereas 10.0 wt% causes severe mechanical degradation, establishing 5.0 wt% as the critical physical saturation threshold. While synthesizing the dense intermediate gradient (1.0–4.0 wt%) fell outside the immediate scope of this primary study, precisely pinpointing the optimal loading boundary within this window remains the main objective of follow-up work.

## 4. Conclusions

In summary, this study successfully developed high-performance PVA/fLGAS composite films via a green, chemical-free aqueous solution casting route without toxic synthetic crosslinkers. Enzymatic modification provided a sustainable route for biowaste valorization while effectively tailoring the side-chain structures of lignin, thereby significantly enhancing its compatibility and uniform dispersion within the PVA matrix. Structural and mechanical evaluations demonstrated that the composite properties are governed by the balance between PVA crystallization and hydrogen bonding. The incorporation of 5 wt% fLGAS constructed a strong physical cross-linking network that yielded a 25% improvement in mechanical performance while maintaining high surface wettability (water contact angle down to 0°) and controlled swelling capabilities (>250%). This confirms that green enzymatic modification can overcome the typical mechanical deterioration associated with biowaste filler incorporation.

This work provides a scalable and sustainable strategy for valorizing industrial lignin waste into high-value functional biopolymer films, reducing reliance on petroleum-derived polymers. While this study establishes the fundamental structure–property relationships and fluid-interaction behaviors of these uncrosslinked biocomposites, future efforts can focus on integrating bio-based functional agents to further expand their practical utility in advanced functional bioplastics.

## Figures and Tables

**Table 1:** The composition of PVA/fLGAS composite films.

Name	PVA (wt%)	fLGAS (wt%)	Distilled Water (wt%)
P-0fLGAS	100	0	10
P-0.1fLGAS	99.9	0.1	10
P-0.5fLGAS	99.5	0.5	10
P-5fLGAS	95.0	5.0	10
P-10fLGAS	90.0	10.0	10

**Table 2:** XRD peak positions (2*θ*) and calculated interplanar spacings (d-spacing) for PVA/fLGAS films.

Sample	Peak Position (2*θ*)	d-Spacing (Å)
P-0fLGAS	19.94	4.45
P-0.1fLGAS	19.67	4.51
P-0.5fLGAS	19.73	4.50
P-5fLGAS	19.73	4.50
P-10fLGAS	19.78	4.49

**Table 3:** Quantitative hydrogen-bonding analysis (FTIR) and degree of crystallinity (Xc, DSC) of PVA/fLGAS composite films.

Sample	Overall Peak Max (cm^−1^)	Cyclic OH (~3220 cm^−1^) Area (%)	OH⋯ether (~3320 cm^−1^) Area (%)	Self-Associated OH (~3420 cm^−1^) Area (%)	*Xc* (%)
P-0fLGAS	3268	24.21	61.77	14.02	34.98
P-0.1fLGAS	3268	22.33	63.46	14.21	31.83
P-0.5fLGAS	3267	20.83	64.79	14.38	28.78
P-5fLGAS	3255	20.56	64.96	14.48	25.25
P-10fLGAS	3248	8.46	73.35	18.19	24.00

**Table 4:** DMA mechanical analysis results of PVA/fLGAS composite films.

Name	Young’s Modulus (MPa)	Yield Stress (MPa)	Yield Strain (%)
P-0fLGAS	2605.7 ± 282	7.73 ± 0.68	0.31 ± 0.02
P-0.1fLGAS	2986.9 ± 203	12.2 ± 3.46	0.41 ± 0.10
P-0.5fLGAS	3023.7 ± 232	11.09 ± 0.95	0.37 ± 0.05
P-5fLGAS	3293.2 ± 274	11.22 ± 2.24	0.33 ± 0.02
P-10fLGAS	1790.2 ± 273	4.23 ± 1.98	0.25 ± 0.13

**Table 5:** Comparison of Young’s modulus between PVA/fLGAS composite film and other reported biopolymer-based composite systems.

Materials	Young’s Modulus (MPa)	Ref.
Lignin, Starch	1992	[36]
PVA, cellulose nanocrystals	31	[37]
PVA, sodium alginate	1916	[38]
PVA, lignin	252	[39]
PVA, fLGAS	3293	This work

## Data Availability

The original contributions presented in this study are included in the article/Supplementary Material. Further inquiries can be directed to the corresponding authors.

## Supplementary Materials

The following supporting information can be downloaded at: https://www.mdpi.com/article/10.3390/polym18182238/s1, Figure S1: FTIR spectra of fLGAS and kLGAS; Figure S2: SEM of (a) P-0fASLG; (b) P-0.1fASLG; (c) P-0.5fASLG; (d) P-5fASLG; (e) P-10fASLG.
