# Supplementary material for: Balancing Hydrogen Bonding and Crystallinity in Enzymatically Functionalized Lignin–Poly(vinyl alcohol) Composite Films
Source: Polymers (Basel). 2026 Sep 14;18(18):2238. doi: 10.3390/polym18182238 (PMC13611349; doi:10.3390/polym18182238)
Supplement: Supplementary file 1 [file polymers-18-02238-s001.zip › polymers-4506066-supplementary.pdf]

Supplementary Materials

Balancing Hydrogen Bonding and Crystallinity in Enzymatically Functionalized Lignin–Poly(vinyl alcohol) Composite Films

Weijun Liang [1], Gabriela Dominguez [2], Miguel Panizo-Laiz [3], Olga Martin [1*] and Alberto García-Peñas [1*]

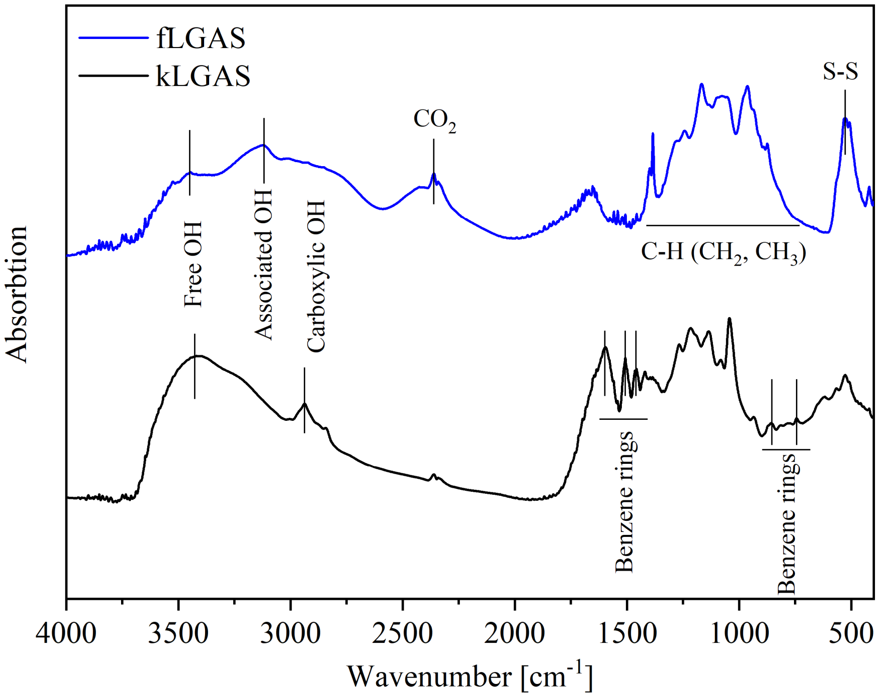

Figure S1. FTIR spectra of fLGAS and kLGAS.

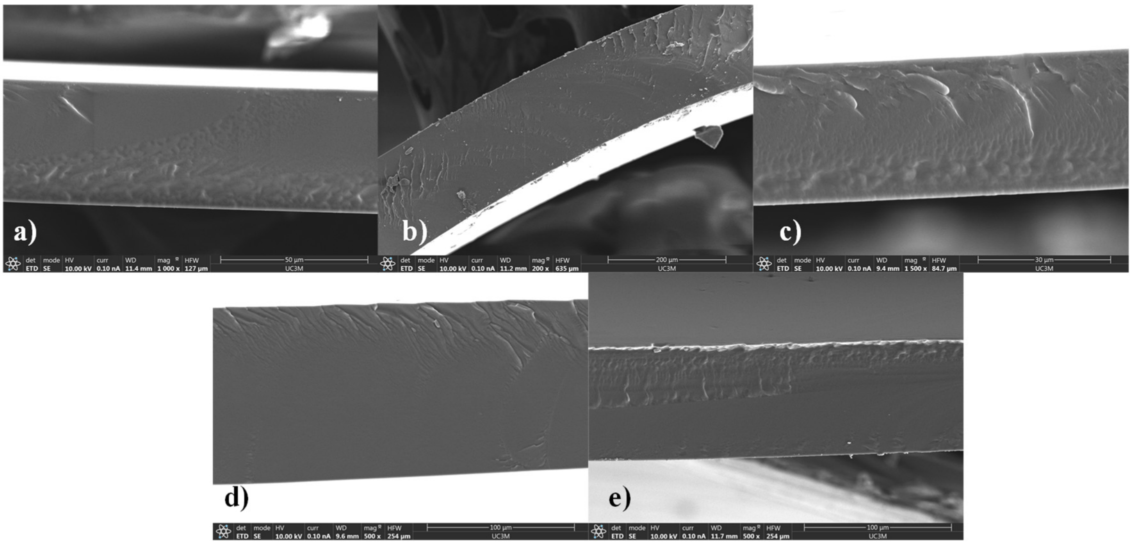

Figure S2. SEM of a) P-0fASLG; b) P-0.1fASLG; c) P-0.5fASLG; d) P-5fASLG; e) P-10fASLG.
